# Reference Values for Spirometry in Moroccan Adults

**DOI:** 10.7759/cureus.61095

**Published:** 2024-05-26

**Authors:** Khalid Bouti, Jouda Benamor, Jamal Eddine Bourkadi, Sanaa Hammi

**Affiliations:** 1 Department of Pulmonology, Mohammed VI University Hospital, Tangier, MAR; 2 Laboratory of Life and Health Sciences, Faculty of Medicine and Pharmacy of Tangier, Abdelmalek Essaâdi University, Tangier, MAR; 3 Department of Pulmonology, Moulay Youssef University Hospital, Rabat, MAR; 4 Faculty of Medicine and Pharmacy, Mohammed V University, Rabat, MAR

**Keywords:** gli 2012, morocco, adults, prediction equations, reference values, spirometry

## Abstract

Introduction: This novel study aimed to establish spirometric reference values and prediction equations based on a sample of the adult Moroccan population, an endeavor that has not been attempted previously.

Methods: In this cross-sectional study involving healthy Moroccan adults, data was collected through a mobile spirometry setup.

Results: Our sample comprised 841 healthy adults (384 men and 457 women) aged 18-86 years who underwent spirometry. For both sexes, the Global Lung Function Initiative 2012 equations for Caucasians corresponded the best to the studied sample but were not perfectly compatible.

Conclusion: The spirometric prediction equations established in this study for Moroccan adults aged 18-86 years best represent the Moroccan population. More extensive future studies are needed to enrich the database of reference values and prediction equations derived from our research.

## Introduction

Spirometry is a key element in the assessment of chronic respiratory diseases. It provides accurate diagnosis, guides further investigations, offers an evaluation of disability, and allows for the ongoing monitoring of patients.

The working group of the European Respiratory Society (ERS) on the Global Lung Function Initiative (GLI) has defined the minimum spirometry data required for establishing prediction equations for a population. This data must be derived from a minimum population of 300 healthy volunteers (150 males and 150 females) [1]. In 2012, the GLI published universal prediction equations for spirometric reference values for individuals of both sexes aged 3-95 years [2]. Moroccan data weren't available yet, so they weren't used in the development of the GLI equations.

To fill this gap and respond to the GLI Working Group's recommendation, we conducted the current study, which aims to establish spirometric reference values and prediction equations for adults of both sexes from a sample of the Moroccan population and compare them with the values and equations derived from other populations.

## Materials and methods

This cross-sectional study collected data through mobile spirometry setup. It included healthy volunteers who underwent correctly performed spirometry. The study was approval by the Biomedical Research Ethics Committee of the Faculty of Medicine and Pharmacy in Rabat, Morocco (approval number: CERB/313, issued on 04/11/2013). To satisfy the ERS Working Group on the GLI equations, we targeted a sample size of at least 300 individuals, with at least 150 males and 150 females. All subjects in the study were informed of its purpose and participated voluntarily. The study sample consisted of individuals residing in the Tanger-Tétouan-Al Hoceima region. Before the spirometry test, each participant answered a standardized hetero-questionnaire, which the physician filled out [3].

The inclusion criteria were as follows: a valid questionnaire, correct execution of the spirometry maneuver [4], provision of consent to participate in the study, and Moroccan nationality. The exclusion criteria included the following: smoking, the presence of respiratory symptoms, acute or chronic respiratory or nonrespiratory diseases, and obesity. All volunteers underwent a thorough clinical examination.

We used a portable spirometer, the Spirolab III (Medical International Research Ltd, Rome, Italy). The spirometer system complied with the ATS/ERS spirometry equipment recommendations. Ventilatory flows were measured under ambient conditions (ambient temperature and pressure saturated) and expressed under conditions of vapor-saturated volume at body temperature (body temperature and pressure saturated). The recommended precautions before and during the performance of spirometry were observed.

After data collection, the results were transferred from the WinspiroPRO software (Medical International Research Ltd, Rome, Italy) to Microsoft Excel (Microsoft Corporation, Redmond, Washington, United States) and then processed using IBM SPSS Statistics for Windows, Version 22.0 (Released 2013; IBM Corp., Armonk, New York, United States).

Statistical significance was considered at p < 0.05. The relationship between each anthropometric parameter (gender, age, height, and weight) and the ventilatory variables was studied using simple linear regression. Parameters significantly correlated with anthropometric parameters were further examined using multiple linear regression. If certain parameters were not significant, they were excluded so that the retest included only the significant parameters. In the end, the prediction equations were developed based on the results of the final test.

The same process was carried out for the following spirometric parameters: forced expiratory volume in one second (FEV1), forced vital capacity (FVC), FEV1/FVC ratio, peak expiratory flow (PEF), maximal expiratory flow at 25% of FVC (MEF25), MEF50, MEF75, and MEF between 25% and 75% of FVC (MEF25-75). The first four parameters were selected to present the results, as they are the most commonly used in daily practice. For our sample, we calculated the lower limit of normal and the differences between the theoretical values from various formulas and our values, expressed as z-scores.

## Results

This spirometric study collected data from 841 healthy adults (384 men and 457 women) aged 18-86 years. Table 1 summarizes the anthropometric and spirometric characteristics of the entire sample as well as those of the male and female populations separately.

**Table 1:** Anthropometric and ventilatory characteristics of the adult spirometry sample. FVC: forced vital capacity; FEV1: forced expiratory volume in one second; PEF: peak expiratory flow; MEF25: maximal expiratory flow at 25% of FVC; MEF50: maximal expiratory flow at 75% of FVC; MEF75: maximal expiratory flow at 75% of FVC; MEF25-75: MEF between 25% and 75% of FVC

Parameters	Total Participants (N = 841)		Male (N = 384)		Female (N = 457)	
	Mean ± SD	Range	Mean ± SD	Range	Mean ± SD	Range
Age (years)	35.2 ± 15.7	18.0–86.9	36.5 ± 15.8	18.00–86.94	34.1 ± 15.7	18.0–85.9
Height (cm)	166.6 ± 9.7	137.0–195.0	173.8 ± 7.2	149.0–195.0	160.5 ± 6.96	137.0–182.0
Weight (kg)	69.7 ± 15.4	30.0–106.0	71.7 ± 11.56	41.0–106.0	60.54 ± 9.56	30.0–91.0
BMI (kg/m²)	25.1 ± 5.0	14.5–29.98	24.9 ± 4.7	14.53–29.98	23.39 ± 3.38	15.4–29.90
FVC	3.85 ± 1.08	0.99–8.21	4.62 ± 0.99	1.45–8.21	3.20 ± 0.64	0.99–5.14
FEV1	3.33 ± 0.89	0.86–6.25	3.92 ± 0.84	1.45–6.25	2.83 ± 0.56	0.86–4.55
FEV1/FVC	87.7 ± 7.5	65–100	85.7 ± 7.8	67.6–100.0	89.4 ± 6.9	65.0–100.0
PEF	7.24 ± 2.12	2.19–14.51	8.84 ± 1.822	3.91–14.51	5.90 ± 1.25	2.19–10.33
MEF25	6.37 ± 1.75	2.13–12.71	7.53 ± 1.64	3.34–12.71	5.39 ± 1.13	2.13–9.44
MEF50	4.49 ± 1.34	1.02–8.80	5.08 ± 1.45	1.18–8.80	4.00 ± 0.99	1.02–7.34
MEF75	2.26 ± 0.95	0.39–6.15	2.44 ± 1.07	0.44–6.15	2.11 ± 0.81	0.39–5.06
MEF25-75	4.16 ± 1.26	0.87–8.96	4.65 ± 1.40	1.33–8.96	3.75 ± 0.95	0.87–6.5

Notably, on average, the men had higher ages, heights, and weights than the women. In contrast, the average body mass index (BMI) of the women was higher than that of the men. Furthermore, the FEV1/FVC ratios were higher in the women, while all spirometric parameters were higher in the men.

**Figure 1:**
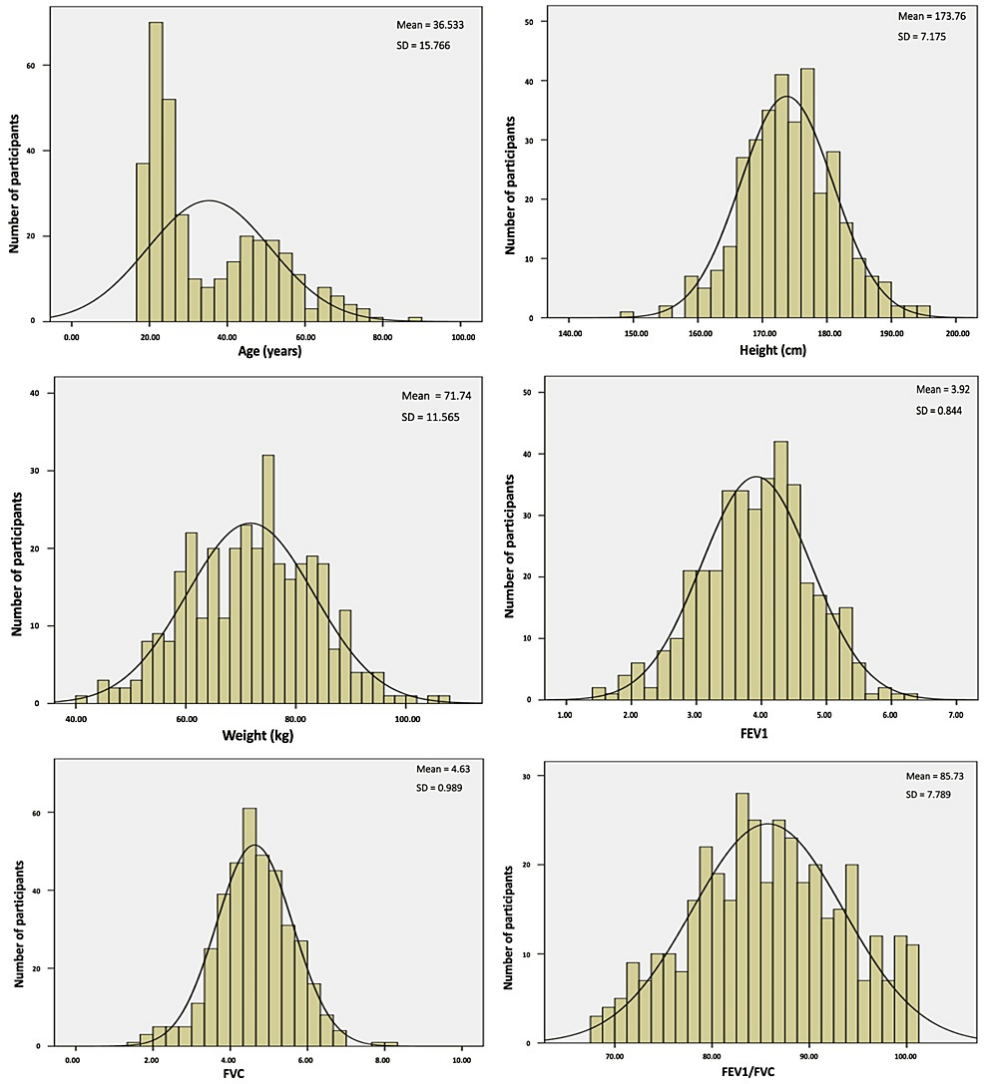
Distribution curves of various anthropometric parameters (age, height, and weight) and ventilatory parameters (FEV1, FVC, and FEV1/FVC) for males. FVC: forced vital capacity; FEV1: forced expiratory volume in one second

**Figure 2:**
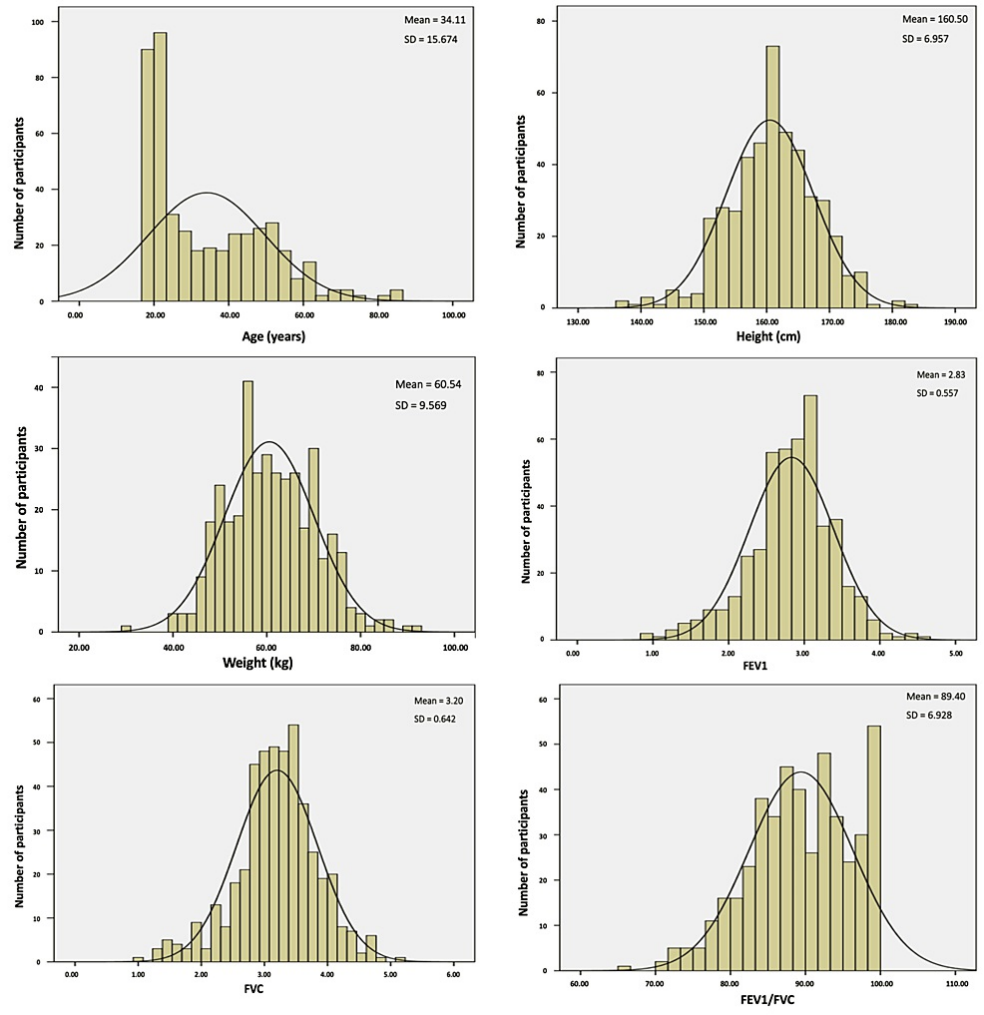
Distribution curves of various anthropometric parameters (age, height, and weight) and ventilatory parameters (FEV1, FVC, and FEV1/FVC) for females. FVC: forced vital capacity; FEV1: forced expiratory volume in one second

The distribution curves of various anthropometric parameters (age, height, weight, and BMI) and ventilatory parameters (FEV1, FVC, and FEV1/FVC) in the sample showed Gaussian distribution for males and females, justifying the use of parametric tests with this sample (Figures 1, 2).

Table 2 shows the sample’s predictive regression models for the main spirometric parameters. All equations depended on age and height. Age had a negative influence on the values of various parameters, whereas height had a positive influence on all parameters, except for the ratios.

**Table 2:** Predictive regression models for various spirometric parameters in Moroccan adults. R: multiple correlation coefficient; R2: coefficient of determination; SEE: standard error of the estimate; RSD: residual standard deviation; FVC: forced vital capacity; FEV1: forced expiratory volume in one second; PEF: peak expiratory flow; MEF25: maximal expiratory flow at 25% of FVC; MEF50: maximal expiratory flow at 75% of FVC; MEF75: maximal expiratory flow at 75% of FVC; MEF25-75: MEF between 25% and 75% of FVC

Gender	Variables	Regression parameters			R	R²	SEE	RSD
		Constant	Age	Height				
Male	FVC	-7.639	-0.022	0.075	0.75	0.56	0.657	0.655
	FEV1	-4.403	-0.029	0.054	0.81	0.66	0.490	0.489
	FEV1/FVC	132.731	-0.193	-0.230	0.37	0.14	7.244	7.225
	PEF	-6.859	-0.025	0.095	0.49	0.24	1.590	1.586
	MEF25	-3.884	-0.027	0.071	0.47	0.22	1.448	1.444
	MEF50	0.191	-0.044	0.037	0.56	0.32	1.205	1.202
	MEF75	1.051	-0.036	0.016	0.58	0.33	0.878	0.875
	MEF25-75	0.796	-0.046	0.032	0.59	0.35	1.129	1.126
Female	FVC	-3.913	-0.014	0.047	0.71	0.51	0.451	0.450
	FEV1	-2.200	-0.018	0.035	0.77	0.59	0.359	0.358
	FEV1/FVC	133.558	-0.148	-0.244	0.34	0.12	6.531	6.517
	PEF	-3.188	-0.006	0.058	0.35	0.12	1.173	1.170
	MEF25	-1.827	-0.011	0.047	0.37	0.14	1.055	1.053
	MEF50	0.718	-0.026	0.026	0.50	0.25	0.859	0.857
	MEF75	2.633	-0.030	0.003	0.59	0.35	0.651	0.650
	MEF25-75	1.352	-0.031	0.022	0.58	0.34	0.776	0.774

**Figure 3:**
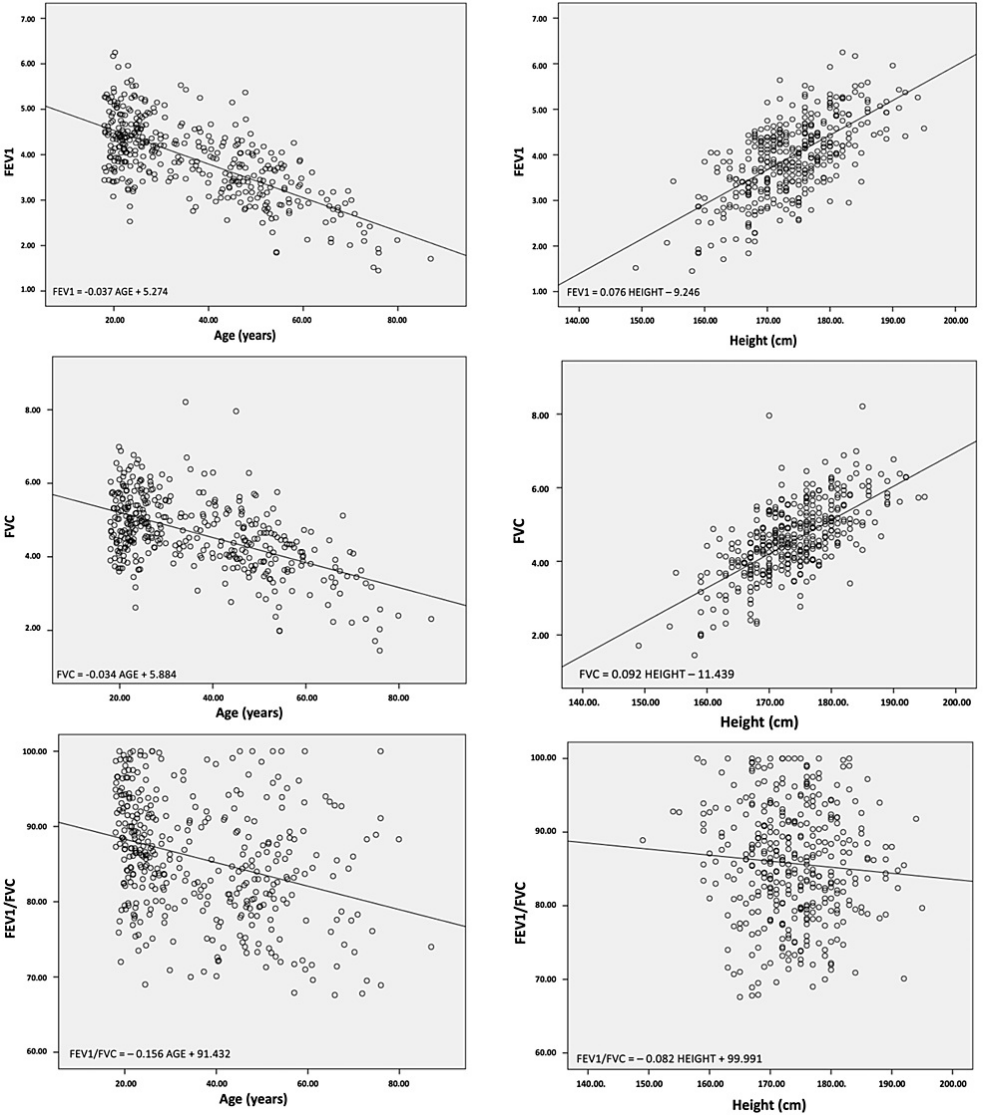
Scatterplots of the FEV1, FVC, and FEV1/FVC values in correlation with age and height (males). FVC: forced vital capacity; FEV1: forced expiratory volume in one second

**Figure 4:**
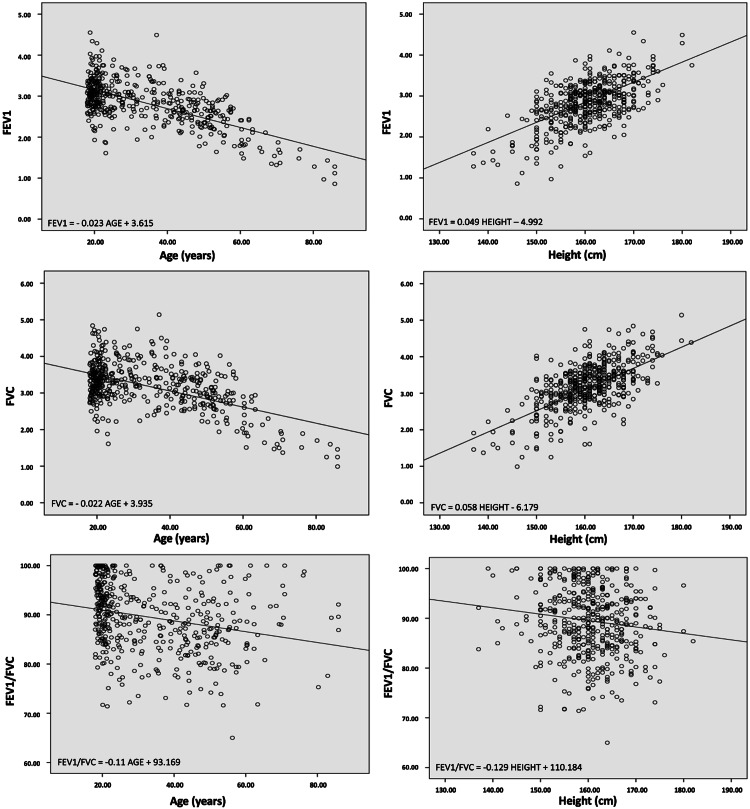
Scatterplots of the FEV1, FVC, and FEV1/FVC values in correlation with age and height (females). FVC: forced vital capacity; FEV1: forced expiratory volume in one second

The scatterplots of the FEV1, FVC, and FEV1/FVC values in correlation with age and height in the sample indicated that the FEV1 and FVC values decreased with age and increased with height (Figures 3, 4). Additionally, the values of the FEV1/FVC ratio showed a negative trend with both age and height.

Summary of equations

The equations derived from the study sample (age in years and height in centimeters) are given below.

For Men Aged 18-86 Years (N = 384)

$FVC = &minus;7.639 &minus; 0.022 Age + 0.075 Height$

$FEV1 = &minus;4.403 &minus; 0.029 Age + 0.054 Height$

$FEV1/FVC = 132.731 &minus; 0.193 Age &minus; 0.230 Height$

$PEF = &minus;6.859 &minus; 0.025 Age + 0.095 Height$

$MEF25 = &minus;3.884 &minus; 0.027 Age + 0.071 Height$

$MEF50 = 0.191 &minus; 0.044 Age + 0.037 Height$

$MEF75 = 1.051 &minus; 0.036 Age + 0.016 Height$

$MEF25-75 = 0.796 &minus; 0.046 Age + 0.032 Height$

For Women Aged 18-85 Years (N = 457)

$FVC = &minus;3.913 &minus; 0.014 Age + 0.047 Height$

$FEV1 = &minus;2.200 &minus; 0.018 Age + 0.035 Height$

$FEV1/FVC = 133.558 &minus; 0.148 Age &minus; 0.244 Height$

$PEF = &minus;3.188 &minus; 0.006 Age + 0.058 Height$

$MEF25 = &minus;1.827 &minus; 0.011 Age + 0.047 Height$

$MEF50 = 0.718 &minus; 0.026 Age + 0.026 Height$

$MEF75 = 2.633 &minus; 0.030 Age + 0.003 Height$

$MEF25-75 = 1.352 &minus; 0.031 Age + 0.022 Height$

## Discussion

The current study provided spirometric reference values and prediction equations for the adult Moroccan population aged 18-86 years; 18-86 for men and 18-85 for women. Although the study subjects were selected from the population through purposeful sampling rather than random selection to cover all age and size ranges for both sexes, they were sourced from a large, sociodemographically representative region of Morocco. Thus, this sample may be representative of the Moroccan population. Published studies have found genetic similarities between the two major ethnicities in Morocco, the Arabs and the Amazigh [5]. However, the single-region nature of the study may introduce selection bias.

The population included only nonsmoker subjects with apparent good health. As all spirometry measures were performed or supervised by the study’s pulmonologist with a focus on quality and as 64 spirometry tests were excluded, it is legitimate to assert that all tests met current ATS/ERS standards.

Given the lack of prior data on spirometric prediction equations for Moroccan subjects, the current results provide the first reliable and reproducible models for this population. Our spirometric study on adults took place in cities in the Tanger-Tétouan-Al Hoceima region. The majority of similar studies have been conducted in single cities, such as Tabuk in Saudi Arabia, Sousse in Tunisia, Constantine in Algeria, Gothenburg in Sweden, Kolkata in India, Ontario in Canada, and Salt Lake City in the United States [6-11]. Studies conducted in multiple centers across several cities such as those of Hankinson during the Third National Health and Nutrition Examination Survey (NHANES III) in the United States, Dockery in the United States, Brandli in Switzerland, and Bashir in Sudan [12-15] are less common.

The size of our sample appeared sufficient, with 841 volunteers, including 457 (54.3%) women and 384 (45.6%) men. Male predominance was observed in several previous studies, including those of El Attar et al. in Tunisia (1,088 men and 104 women) [16] and Morris et al. in the United States (509 men and 454 women) [17]. Some study samples were exclusively composed of men, such as those of Louw et al. in South Africa (208 men) [18], Singh et al. in Malaysia (1,485 men) [19], and Sharp et al. in the United States (528 men) [20].

Similar to our study, several previous research works have had a female predominance, such as those of Eom and Kim in South Korea (4,047 women and 706 men) [21] and Tan et al. in Canada (927 women and 729 men) [22]. Only Boutros-Toni’s study had an exclusively female sample, consisting of 290 adolescent girls and women aged 10-70 years [23]. Finally, Sirotkovic and Cvorisćec’s study included an equal number of men (n=1,250) and women (n=1,250) [24], as did Belacy et al.’s work in Saudi Arabia (250 men and 250 women) [6].

Future studies should further improve the accuracy of our prediction equations by utilizing similarly large samples in all other regions of Morocco. We cannot propose extrapolating these reference equations beyond the age range studied during the development of this Moroccan data. We tested various statistical models, but simple and multiple linear regression models were the most coherent and provided the best correlations. In adults aged 18-86 years, the values of the parameters regress with age, unlike in children and adolescents, where lung capacities develop due to the growth of the lungs and the musculature involved in breathing.

All equations derived from this study have gender, age, and height as independent variables. This is the case in most published studies, such as those of Ratomaharo et al. in Madagascar [25], Roberts et al. in England [26], Schwartz et al. (NHANES II) in the United States [27], Tabka et al. in Tunisia [28], Ben Saad et al. in Tunisia [7], and Quanjer et al. with their multiethnic 2012 equations [2]. Other studies have considered weight, gender, age, and height as independent variables, such as those of Etemadinezhad and Alizadeh in Iran [29], Mu and Liu in China [30], Hedenstrom et al. in Sweden [31], and La Paglia et al. in Sicily [32].

Currently, the FEV1 of Moroccans can be predicted most accurately using the GLI prediction equations for Caucasians and the NHANES III for Mexicans in men as well as those of the GLI for Caucasians and Northeast Asians and the NHANES III for Mexicans in women. Moroccans’ FVC can be predicted most accurately using the prediction equations of the GLI for Caucasians and the NHANES III for Caucasians and Mexicans in men as well as those of the GLI for Caucasians and Northeast Asians and the NHANES III for Caucasians and Mexicans in women. Finally, the FEV1/FVC ratio of Moroccans can be predicted most accurately overall by the GLI prediction equations for Southeast Asians for both men and women.

Our equations accurately represent Moroccan spirometric values. While awaiting the implementation of these equations for adults, the GLI 2012 prediction equations for Caucasians correspond best to Moroccan subjects.

The GLI 2012 equations were derived from over 160,000 data points from 72 centers in 33 countries [2]. After applying inclusion and exclusion criteria, 97,759 records of healthy nonsmokers (55.3% women) aged 2.5-95 years were retained. After 23,572 records were rejected, primarily because they could not be combined with other ethnic or geographic groups, reference equations were derived for healthy individuals aged 3-95 years for Caucasians (n=57,395), African Americans (n=3,545), Northeast Asians (n=4,992), and Southeast Asians (n=8,255). Lung function data was collected, and prediction equations were derived using the generalized additive model for location, scale, and shape. For individuals not represented by these four groups or of mixed ethnic origins, a composite equation derived from the average of the above equations was provided for interpretation until a more appropriate solution was developed. Additional data from the Indian subcontinent and Arab countries as well as from Polynesia, Latin America, and Africa was actively sought, which is what motivated our study.

The spirometric reference values for Caucasian, African American, and Mexican American adults aged 8-80 years were developed from data collected from 7,429 asymptomatic, nonsmoking participants in the NHANES III [12]. The Caucasian subjects had higher average FVC and FEV1 values than the Mexican American and African American subjects across all age groups. The reference values and lower limits of normal were obtained using a piecewise polynomial model with age and height as predictors.

In this study, we compared the z-scores of the FEV1, FVC, and the FEV1/FVC ratio for both sexes using our equations and other published reference values. If we sum the z-scores, we will have a specific cumulative z-score for each equation. If we divide each cumulative z-score by six, we will have a specific average z-score for each equation (Table 3).

**Table 3:** Simple, cumulative, and average z-scores resulting from the comparison of values from our equations with values from various international equations studied for FEV1, FVC, and FEV1/FVC in adults of both sexes. GLI: Global Lung Function Initiative; ECSC: European Community for Steel and Coal; NHANES III: Third National Health and Nutrition Examination Survey; FVC: forced vital capacity; FEV1: forced expiratory volume in one second

		GLI					ECSC	NHANES III		
		Caucasians	African Americans	Northeast Asians	Southeast Asians	Other		African Americans	Caucasians	Mexicans
Male	FEV1	-0.18	-0.84	-0.34	-0.56	-0.49	-0.4	-0.84	-0.47	-0.02
	FVC	0.11	-0.76	-0.23	-0.56	-0.41	-0.28	-0.69	0.06	0.05
	FEV1/FVC	-0.47	-0.34	-0.41	-0.15	-0.34	-0.78	-0.48	-0.75	-0.56
Female	FEV1	-0.1	-0.78	-0.18	-0.69	-0.44	-0.49	-0.89	-0.47	-0.09
	FVC	0.12	-0.61	0	-0.59	-0.28	-0.26	-0.68	0.17	0.18
	FEV1/FVC	-0.78	-0.71	-0.64	-0.42	-0.64	-1.22	-0,85	-0.87	-0.87
Cumulative z-score		-1.3	-4.04	-1.8	-2.97	-2.6	-3.43	-4.43	-2.33	-1.31
Average z-score		-0.216	-0.673	-0.300	-0.495	-0.433	-0.571	-0.738	-0.388	-0.218

The study's findings have limitations stemming from its focus on a single region within Morocco, a country of 12 diverse regions. Additionally, the participant pool includes only two of the four known ethnicities in Morocco. These factors suggest potential regional and ethnic variations in lung function that were not captured in the current data. To create a more comprehensive understanding, the study recommends expanding research to include adults from other regions and ethnic backgrounds, enriching the database of reference values and prediction equations.

To address the potential influence of confounding variables, such as regional and ethnic differences, we employed statistical techniques to control for these factors during data analysis, ensuring a more accurate assessment of lung function across diverse populations. However, while awaiting further studies, we recommend utilizing the study results within the two ethnicities, the specified age range, and the region covered by the study.

## Conclusions

The current study established spirometric prediction equations for Moroccan adults aged 18-86 years. These equations best represent the Moroccan adults. Currently, while awaiting the implementation of our equations for adults, the GLI 2012 prediction equations for Caucasians correlate best with Moroccan subjects. Future studies are required to enrich the database of reference values and prediction equations derived from our research.
